# Performance of Claims-Based Frailty Measures in Medicare FFS and MA: Findings from NHATS

**DOI:** 10.1093/geroni/igaf122.4389

**Published:** 2025-12-31

**Authors:** Lan Luo, Xiecheng Chen, Chan Mi Park, Sandra Shi, Kevin Pritchard, Ellen McCarthy, Dae Hyun Kim

**Affiliations:** Hebrew SeniorLife, Boston, Massachusetts, United States; Hebrew Rehabilitation Center, Roslindale, Massachusetts, United States; Hebrew SeniorLife, Harvard Medical school, Boston, Massachusetts, United States; Hebrew SeniorLife, Boston, Massachusetts, United States; Hebrew SeniorLife, Roslindale, Massachusetts, United States; Hebrew SeniorLife, Boston, Massachusetts, United States; Hebrew SeniorLife, Boston, Massachusetts, United States

## Abstract

Frailty is a state of vulnerability in older adults which predicts adverse health outcomes. Although clinical measures, such as a Comprehensive Geriatric Assessment–Frailty Index (CGA-FI) and the Fried phenotype (FP), are reference standards, claims-based measures are often used for research. Given the rapidly growing Medicare Advantage (MA) population and concerns regarding MA data completeness, we assessed performance of claims-based frailty models in detecting frailty by CGA-FI and FP among community-dwelling MA and fee-for-service (FFS) beneficiaries. We measured the Kim’s Claims-based Frailty Index (CFI) and Gilbert’s Hospital Frailty Risk Score (HFRS) in the National Health and Aging Trends Study linked to Medicare claims. In 2019 (FFS n = 2,153, MA n = 1,672), CFI had high specificity (FFS 0.97, MA 0.99) but low sensitivity (FFS 0.31, MA 0.24) in detecting frailty by CGA-FI. The CFI’s PPV was higher for CGA-FI than FP (FFS: 0.84 vs 0.44, MA: 0.90 vs 0.47). In comparison, HFRS had high sensitivity (FFS 0.91, MA 0.91) but low specificity (FFS 0.38, MA 0.43) for CGA-FI frailty, with similar results for FP. Model discrimination was good (C-statistics: CFI 0.81, HFRS 0.79), with both models performing better when benchmarked against CGA-FI. 2021 analyses were similar. In sensitivity analysis using only inpatient claims, HFRS sensitivity decreased while specificity increased. These results suggest that claims-based frailty measures may perform comparably in MA and FFS beneficiaries. However, performance characteristics between models vary – highlighting the importance of model selection for a given population or purpose.

